# BRCA2 and Other DDR Genes in Prostate Cancer

**DOI:** 10.3390/cancers11030352

**Published:** 2019-03-12

**Authors:** Paz Nombela, Rebeca Lozano, Alvaro Aytes, Joaquin Mateo, David Olmos, Elena Castro

**Affiliations:** 1 Prostate Cancer Clinical Research Unit, Spanish National Cancer Research Center, 28029 Madrid, Spain; mpnombela@ext.cnio.es (P.N.); rlozano@ext.cnio.es (R.L.); dolmos@cnio.es (D.O.); 2 CNIO-IBIMA Genitourinary Cancer Research Unit, Institute of Biomedical Research in Malaga (IBIMA), 29010 Málaga, Spain; 3 Programs of Molecular Mechanisms and Experimental Therapeutics in Oncology (ONCOBell), and Cancer Therapeutics Resistance (ProCURE), Catalan Institute of Oncology, Bellvitge Institute for Biomedical Research, L’Hospitalet de Llobregat, 08908 Barcelona, Spain; aaytes@idibell.cat; 4 Vall d’Hebron Institute of Oncology, Vall d’Hebron University Hospital, 08035 Barcelona, Spain; jmateo@vhio.net; 5 Medical Oncology Department, Hospital Universitario Quironsalud Madrid, 28223 Madrid, Spain

**Keywords:** *BRCA2*, DNA damage and repair, DDR, prostate cancer, PARP inhibitors

## Abstract

Germline and somatic aberrations in DNA damage repair (DDR) genes are more prevalent in prostate cancer than previously recognized, with *BRCA2* as the most commonly altered gene. Germline mutations in *BRCA2* have been linked to poor prognosis when patients are managed under the protocols currently approved for prostate cancer. The impact of germline mutations in other DDR genes beyond *BRCA2* remain unclear. Importantly, a quarter of prostate cancer patients identified as germline mutation carriers lack a family history of cancer. The clinical implications of somatic DDR defects are yet to be elucidated. Poly ADP-ribose polymerase (PARP) inhibitors and platinum-based chemotherapy have proven to be effective in the treatment of other tumor types linked to *BRCA1* and *BRCA2* alterations and several trials are currently evaluating their efficacy in prostate cancer. Here, we summarize the available evidence regarding the prevalence of somatic and germline DDR defects in prostate cancer; their association with clinical outcomes; the trials assessing the efficacy of new therapies that exploit DDR defects in prostate cancer and briefly discuss some uncertainties about the most appropriate management for these patients.

## 1. Introduction

Alterations in the DNA damage repair (DDR) pathways have only recently been recognized as a major hallmark of prostate cancer. Next-generation sequencing studies have revealed that about 10% of primary tumors and 25% of metastases from prostate cancer harbor DDR defects [1,2] with *BRCA2* aberrations consistently described as the most common event. Germline deleterious mutations in DDR genes are present in 8–16% of metastatic prostate cancer patients [1,3,4], a prevalence significantly higher than previously recognized. Inherited *BRCA2* mutations that impair the gene function have been described in 3–5% of patients with advanced prostate cancer [3,4]. These *BRCA2* mutations have been associated with more aggressive disease and poor clinical outcomes [5,6,7,8,9], but the prognostic implications of other DDR genes are less well established. On the other hand, there is strong emerging evidence that some germline and somatic DDR defects may predict the response to poly-ADP ribose polymerase (PARP) inhibitors and platinum-based chemotherapy in prostate cancer [10,11,12,13]. These findings make genetic testing attractive not only for risk stratification, but also for treatment selection. The decrease in the cost of sequencing and the broad access to these platforms will presumably result in an increased number of prostate cancer patients identified as DDR mutation carriers, who could benefit from appropriate genetic counseling and personalized management and therapies.

## 2. Alterations in DNA Repair Genes Are Common in Prostate Cancer

Several recent studies cataloguing the genetic landscape of prostate cancer have shown that a significant proportion of cases harbor aberrations in the DDR genes [1,2,14,15,16,17].

Nineteen per cent of the 333 primary prostate tumors sequenced by The Cancer Genome Atlas Research Network (TCGA) had deleterious germline or somatic aberrations in the DDR genes including *BRCA2*, *BRCA1*, *CDK12*, *ATM*, *FANCD2*, and *RAD51C* [16]. However, all six cases with germline *BRCA2* mutations presented the same variant, p.K3326* (c.9976A>T), arguably deleterious. The International Stand Up to Cancer/Prostate Cancer Foundation/American Association for Cancer Research Prostate Cancer Team identified alterations in the DDR genes in 23% of the 150 metastatic biopsies analyzed [1]. *BRCA2* was altered in 13% of samples followed by *ATM* (7.3%), *MSH2* (2%), and *BRCA1*, *FANCA*, *MLH1*, *RAD51B*, and *RAD51C* (0.3%). A larger study that analyzed 680 primary tumors and 333 metastatic samples including cases from the previously mentioned studies, identified germline and/or somatic DDR defects in 10% and 27% of the primary and metastatic samples, respectively [2].

The study by Robinson et al. [1] provided the first evidence suggesting that germline mutations in DDR genes known to be linked to increased cancer risk were present in metastatic prostate cancer with a higher prevalence than previously recognized. Unexpectedly, 8% of the DDR mutations identified in the metastatic samples were in the germline. In 2016, a study of germline mutations in 692 men with metastatic prostate cancer revealed that 11.8% of them harbored a germline mutation in one of the 20 DDR genes associated with the cancer-predisposition syndromes analyzed [3] (Table 1). The fact that this prevalence was significantly higher than the 5% identified in men with localized disease and the 3% in the general population [3,16], suggests that such events may predispose men to aggressive forms of the disease. The PROREPAIR-B study screened 419 unselected men in Spain with metastatic prostate cancer for germline mutations in 107 genes (Table 1) and found that 7.4% of the participants carried inherited mutations in any of the genes studied by Pritchard et al. [3,4]. The variation in prevalence was likely to due to the different genetic background of both populations. In the series reported by Pritchard et al., Ashkenazi founder mutations *BRCA1* c.5266dupC and *BRCA2* c.5946delT accounted for 66% and 24% of the mutations identified in *BRCA1* and *BRCA2*, respectively. Similarly, the Eastern European founder mutation *CHEK2* c.1100del represented 50% of all mutations in *CHEK2*. These three mutations, which accounted for 22% of all mutations, are very rare in the Spanish population included in PROREPAIR-B [18,19] and no carriers of these variants were identified. However, *BRCA2* remained the most frequently mutated gene in this second study, although with a lower prevalence (3.3%) than the one previously reported (5.3%). It is also possible that in the time elapsed between the two studies, some variants initially considered as likely pathogenic have been reclassified as variants of unknown significance, resulting in a lower prevalence of pathogenic mutations in the second study. Screening studies in groups with different genetic backgrounds and an accurate classification of genetic variants are needed for a more precise estimation of the prevalence of germline mutations in DDR genes across populations.

## 3. Impact of BRCA Mutations on Clinical Outcomes and Response to Treatment in Prostate Cancer

### 3.1. Management of Localized Disease

A range of management options is available for men with localized prostate cancer. The criteria to consider active surveillance, radiotherapy, or radical prostatectomy for the general population are usually based on several factors including PSA levels at diagnosis, Gleason score, tumor stage, performance status, life expectancy, and patient preference [23,24]. *BRCA1* and *BRCA2* mutation carriers with localized disease are usually managed under the same general protocols as no definitive evidence has demonstrated that they should be treated otherwise.

Active surveillance can be an adequate management strategy for men with low-risk localized prostate cancer unlikely to affect the patient’s life expectancy in the absence of treatment [24]. It consists of regular PSA monitoring and MRI, allowing curative intervention for those patients that experience disease progression. The poor outcomes associated with *BRCA2*-related prostate tumors have discouraged physicians to recommend active surveillance to eligible carriers although evidence to support this decision has only been provided recently. A report analyzing the outcomes of 1211 men undergoing active surveillance including 11 *BRCA1*, 11 *BRCA2*, and 5 *ATM* germline carriers showed that tumors in *BRCA2* carriers were more likely to present a tumor grade reclassification requiring radical treatment when compared to non-carriers. The tumor staging upgrade incidence at 2-, 5- and 10- years was 27%, 50%, and 78% in *BRCA2* carriers compared to 10%, 22% and 40% in non- carriers (*p* = 0.001) [25].

No conclusive data are available regarding whether alterations in *BRCA2* or other DDR genes are relevant in selecting between curative treatment options (radical prostatectomy or radiotherapy). The only reported data comes from a retrospective study that analyzed the outcomes of 1200 patients with localized disease including 18 *BRCA1* and 49 *BRCA2* carriers, and reported that 89% and 67% of *BRCA* surgically treated were free from metastasis at 5 and 10 years, respectively, when compared to 97% and 91% of non-carriers (*p* = 0.024). For those treated with radiotherapy, the difference was even greater as only 57% and 39% of *BRCA1/2* carriers were free from metastasis at 5 and 10 years, respectively, compared to 91% and 80% of non-carriers (*p* < 0.001) [9].

One could be tempted to assume that surgery is a better approach for mutation carriers, however, this conclusion cannot be based on this study. The two treatment cohorts were not balanced due to the fact that patients treated with radiotherapy (both carriers and non-carriers) presented with more advanced disease than those men surgically treated, and a direct comparison of the two groups was not performed. Nonetheless, these data raise the question of whether adjuvant treatments may be beneficial for *BRCA1/2* carriers. Androgen deprivation therapy (ADT) is routinely added to radiotherapy for localized disease. No evidence has been produced to support a different ADT scheme in carriers, but since androgen deprivation seems to downregulate both homologous recombination (HR) [26] and non-homologous End Joining (NHEJ) [27,28], prolonged ADT or the addition of new androgen receptor signaling inhibitors (ARSI) such as abiraterone, enzalutamide, or apalutamide, might be of benefit for those carriers undergoing radiotherapy. Adjuvant chemotherapy with docetaxel following radiotherapy in unselected patients with high-risk disease improves relapse-free survival, but the benefit in metastasis free survival and overall survival is less clear [29]. Several trials are currently ongoing that would provide definite data on the impact of adjuvant chemotherapy or ARSI on metastasis free survival and overall survival. To our knowledge, outcome analyses in these studies have not been planned to be stratified by *BRCA* and/or DDR status. However, if conducted, such analyses would provide an insight into the benefit of different adjuvant schemes and guide future clinical trials in this population.

### 3.2. Management of Metastatic Prostate Cancer

After failure of the primary treatment, the disease is usually managed with long-term androgen deprivation therapy (ADT). Disease progression on continuous ADT is termed castration resistant prostate cancer (CRPC). The PROREPAIR-B study has shown that germline *BRCA2* mutation carriers become resistant to ADT faster than non-carriers. In the study, the median time from continuous ADT initiation to CRPC was 28 months for non-carriers when compared to 13.2 months in *BRCA2* carriers (*p* = 0.05) [4].

PROREPAIR-B has been the first prospective study to address the impact of germline mutations in *BRCA1*, *BRCA2*, and other DDR genes in patients with metastatic CRPC (mCRPC) [4]. Although the 10-month difference in cause specific survival (CSS) between *ATM*, *BRCA1*, *BRCA2*, *PALB2* mutation carriers, and non-carriers (33.2 vs. 23) was not statistically significant (*p* = 0.264), the study showed that CSS from mCRPC was halved in *BRCA2* carriers when compared to non-carriers (17 vs. 33, *p* = 0.027). The difference remained significant when the *BRCA2* carriers were compared to other germline DDR carriers (median 33.8 months, *p* = 0.048). Multivariate analyses identified *BRCA2* as an independent prognostic factor for CSS in mCRPC (HR 2.11, 95% CI 1.06–4.18). Importantly, none of the carriers in this series had received a Poly ADP-ribose polymerase (PARP) inhibitor and/or platinum-based chemotherapy, which may have had a confounding effect on survival.

Analysis of the response to taxanes and ARSIs in the PROREPAIR-B study showed no difference in the response rates based on carrier status [4]. However, the duration of the responses tended to be shorter in carriers, particularly in those harboring *BRCA2* mutations. Importantly, the outcomes of *BRCA2* carriers who received abiraterone or enzalutamide as first-line treatment did not differ from that of non-carriers. Conversely, *BRCA2* carriers treated with the taxane-ASI sequence had significantly worse CSS (median 28.4 vs. 10.7 months, *p* = 0.0003; HR:4.16, 95% CI 1.80–9.62) and the progression-free survival from the first systemic therapy until progression on the second systemic therapy (PFS2) was also shorter (median 17.1 vs. 8.6 months, *p* < 0.0001; HR:8.16 95% CI 3.60–18.49) than in non-carriers who received the same treatment [30]. No biomarker has been identified to date for selecting one therapy over another in the setting of advanced prostate cancer. If these preliminary results are confirmed, the determination of germline *BRCA2* status would be of assistance for the selection of the first line of treatment in mCRPC.

The observations described above may contribute to explain the contradictory results previously reported by three retrospective series that have evaluated the response of germline DDR carriers to abiraterone and enzalutamide. Annala et al. [22] analyzed the outcomes of 176 metastatic CRPC patients including 22 DDR carriers (16 *BRCA2*) (Table 1), and found that the progression free survival (PFS) of DDR carriers on first-line ASI was significantly shorter than that of non-carriers (3.3 vs. 6.2 months, *p* = 0.01). The poor PFS could be related to the high tumor burden in the patients included as reflected by the high levels of circulating tumor DNA (ctDNA) reported (>30%). Authors also remarked on the great heterogeneity observed in PFS, with some DDR carriers benefiting from ARSIs for >2 years. This was the main observation of the second study by Antonarakis et al. [21], who observed a trend toward a more prolonged PFS in mutation carriers when compared to non-carriers (15 vs. 10.8 months, *p* = 0.090) treated with ARSi. Interestingly, they also identified previous chemotherapy as a factor associated with worse PFS and CSS, but did not analyze whether this affects the same to carriers and non-carriers. The third study is the retrospective analysis of patients from the landmark publication by Pritchard et al. in 2016 [20]. The authors reported the outcomes of 330 non-carriers and 60 DDR carriers (including 37 *BRCA2*) and found no association between mutation status and the response or duration of treatment to the first ARSI or taxane administered.

The clinical implications of somatic mutations in *BRCA2* and other DDR genes have not been well characterized yet and there is no evidence of whether these patients may respond differently to the treatment options currently available.

## 4. Mutations in BRCA and Other DNA Repair Genes as a Potential Target for Platinum-Based Chemotherapy and PARP Inhibitors in Prostate Cancer

Specific treatment strategies for patients with somatic and/or germline mutations in DNA repair genes could be obtained from research in other tumor types frequently associated with these events such as breast and ovarian cancers. Platinum-based chemotherapy has been proven to be an effective treatment for *BRCA1* and *BRCA2* mutated breast [31,32] and ovarian cancers [33] as these compounds generate DNA cross-links that cannot be easily resolved with an impaired homologous recombination (HR). In standard protocols for prostate cancer, platinum-based chemotherapy is only used when neuroendocrine differentiation has occurred as phase III trials in mCRPC failed to show any benefit in unselected population [34]. However, several retrospective reports have suggested that *BRCA2* mutated prostate cancer may be highly sensitive to this therapy [10,11,12]. In a retrospective analysis of 141 men with mCRPC treated at the Dana Farber Cancer Institute between 2001–2015 with at least two doses of carboplatin and docetaxel, the treatment demonstrated benefits for patients with germline *BRCA2* mutations [12]. Six out of the eight *BRCA2* carriers identified (75%) presented a >50% PSA decline within 12 weeks of initiating this regimen when compared to 23 of 133 of non-carriers (17%) (*p* = 0.001). A >50% PSA decline was associated with a more prolonged survival (18.9 in *BRCA2* carriers vs. 9.5 months in non-carriers). Several studies are ongoing to evaluate the role of platinum-based chemotherapy for patients with DNA repair defects.

Inhibition of the Poly ADP-ribose polymerase (PARP) is another strategy to treat DNA repair deficient tumors as these drugs exploit the dependency of HR-deficient tumors on alternative DDR pathways [35] and several PARP inhibitors are at different stages of clinical development (Table 2). They differ in their potency and specificity to bind PARP and their ability to trap PARP-DNA complexes [36]. Olaparib (AstraZeneca) was the first drug in class to be approved in 2014 for the treatment of ovarian cancer associated with *BRCA* defects. The first-in-man clinical trial of olaparib in a population of patients with advanced solid tumors, enriched for germline *BRCA1* and *BRCA2* mutations included three mCRPC patients; one of them benefited from the drug for over two years [37]. Small numbers of mCRPC patients with germline *BRCA* mutations were also enrolled in phase I trials with other PARP inhibitors such as talazoparib [38] or niraparib as single agents [39].

An open-label single-arm basket phase II study of olaparib for germline *BRCA* mutation carriers included eight mCRPC patients. Of these, one of four and three of four with or without previous exposure to platinum, respectively, achieved a response to therapy [40]. The phase II trial TOPARP-A [13] enrolled 50 men with heavily pre-treated mCRPC. Fourteen out of the 16 patients who were found to harbor a DDR defect (somatic or germline) (88%) achieved clinical benefit (including radiological responses, PSA drops, and/or CTC count decreases) and durable responses to the PARP inhibitor olaparib including all seven patients with *BRCA2* defects, but also some with *BRCA1, ATM, PALB2*, and *FANCA* defects, among others. The preliminary results of the phase II trial TRITON2 evaluating the efficacy of rucaparib in a preselected population with DDR defects in tumor or ctDNA showed PSA and radiographic responses in 48% and 45% of patients harboring *BRCA2* defects. Confirmed PSA and radiographic responses were also observed in one patient with *BRIP1* and one with *FANCA* mutations [41]. No confirmed responses were observed for *ATM*. This raises the question of which DDR genes other than *BRCA1* and *BRCA2* may be considered predictive of response to PARP inhibitors. However, this point may not be clarified until phase III trials are completed.

A cross-talk between the androgen receptor (AR) and DNA repair has been extensively described [26,27,42]. First, PARP is involved in androgen dependent transcription and PARP inhibition impairs this process [43]. Second, the androgen receptor regulates the transcription of DNA repair genes and therefore androgen depletion impairs HR [26], so the tumor may become susceptible to PARP inhibition regardless of HR mutation status. Supported by this preclinical data, several trials are addressing the potential synergisms between PARP inhibitors and ARSIs, irrespective of DDR status. The first study reported was NCI9012, a phase II trial that compared the efficacy of veliparib plus abiraterone with abiraterone in monotherapy. No differences in PSA response rate (63.9% vs. 72.4%, *p* = 0.27), radiographic response rate (45% vs. 52.2%, *p* = 0.51), or median PFS (10.1 vs. 11 months, *p* = 0.99) were observed between the two groups, with all patients considered. However, 20 of the 148 (25%) patients included in the study had biallelic DDR defects and presented better PSA (90% vs. 56%, *p* = 0.007) and radiographic (87.5 vs. 38.6, *p* = 0.001) response rates than the DDR-wild type, in both treatment arms. The small number of DDR-defective patients in each treatment arm did not allow further comparisons. The combination was well tolerated. Grade ≥3 side effects were observed in 20% and 24% of the single and combination arms, respectively [44].

A second phase II randomized trial assessed the efficacy and tolerability of olaparib in combination with abiraterone when compared with abiraterone in mCRPC patients, irrespective of their DDR status. Eleven of 71 (15%) men in the olaparib arm and 10 out of 71 (14%) patients in the control arm had mutations in the HR genes. However, 61% of patients only had partially characterized HR status as the results of germline and plasma testing could not be confirmed by tumor analysis. Unlike the previous study, time to radiographic progression was significantly prolonged in the olaparib plus abiraterone group when compared to the abiraterone alone group (13.8 vs. 8.2, *p* = 0.034), regardless of HR status. The study was not powered for subgroup analysis, but the exploratory analysis showed a benefit on time to radiographic progression with the combination in patients with and without HR defects. No differences in radiological response rates or in PSA responses were observed between the two arms. Importantly, 54% of patients in the combination arm presented severe adverse events when compared to 28% in the abiraterone group, including seven (10%) patients with a serious cardiovascular event [45].

The contradictory results of these two studies regarding the benefit of adding a PARP inhibitor to an ARSI in DDR-proficient patients to prolong the time to progression may be related to the different pharmacological activity of veliparib and olaparib, but also to the different classification of patients by DDR-status. Currently ongoing trials are likely to clarify this in the near future as well as whether the benefit may outweigh the potential toxicity of the combinations (Table 2).

Unfortunately, resistance to PARP inhibitors eventually arises, even in patients with *BRCA2* biallelic loss who usually present strong initial responses. Early reports suggest that subclonal aberrations reverting germline and somatic DDR mutations back in frame may be a key mechanism of resistance [46,47]. Likewise, polyclonal *BRCA2* reversion mutations have also been identified at the time of disease progression in a patient treated with platinum-based chemotherapy [48]. In all cases, these subclonal events likely driving resistance to PARP inhibitors and platinum, were identified in circulating tumor DNA (ctDNA), reinforcing the clinical utility of ctDNA as a multipurpose biomarker for treatment with PARP inhibition in mCRPC [47].

## 5. Implications for Hereditary Cancer and Germline Testing

Prostate cancer is one of the most heritable human cancers as 57% of the interindividual variation in risk has been attributed to genetic factors [49]. Men harboring an inherited *BRCA2* mutation have a 30% lifetime-risk of developing prostate cancer, although it may vary from 19% to 61% depending on the presence/absence of genetic variants acting as risk-modifiers [50]. The impact of germline *BRCA1* mutations is more modest as the life-time risk of prostate cancer associated with these mutations has been estimated in 13% [50], similar to that of the general population. The prostate cancer risk for other DDR genes beyond *BRCA 1/2* remains unclear.

A Gleason score ≥8, and nodal and distant metastasis at the time of diagnosis are more common in *BRCA* carriers who develop prostate cancer than in non-carriers [8]. Despite the compelling evidence indicating that *BRCA2* mutations predispose carriers to an aggressive prostate cancer phenotype, the most appropriate screening strategy has yet to be elucidated. International guidelines recommend annual PSA-based prostate cancer screening from the ages between 40–45 [51]. The efficacy of this approach is being analyzed in the IMPACT study. IMPACT is an international multicenter study that has enrolled over 2000 men including 791 *BRCA1* and 732 *BRCA2* carriers aged 40–69 years. Participants have annual PSA tests and the threshold to indicate a biopsy is a PSA >3 ng/mL. Data from the first round of annual PSA screening have estimated the positive predictive value of PSA >3 ng/dL in 48% for *BRCA2* carriers, double than the 24% estimated for the general population [52]. Importantly, most of the tumors in *BRCA2* carriers identified through PSA in this preliminary report are of intermediate or high risk unlike those detected through PSA screening in the general population, who are often low-risk tumors [52]. However, considering the limitations of PSA-only screening in populations at increased risk for prostate cancer, assessing the role of additional imaging and urine or blood biomarkers in *BRCA* carriers would be important. The PRECISION study has demonstrated the value of multiparametric magnetic resonance imaging (mpMRI) as a screening tool for prostate cancer [53] and further studies should now assess if this more precise screening tool is of particular benefit for men at an increased risk of aggressive prostate cancer due to inherited *BRCA* mutations.

Beyond prostate cancer, germline *BRCA1* and *BRCA2* mutations are known to increase the risk of other tumor types including breast and ovarian cancers. More than 25 genes linked to DNA repair have been associated with familial breast and/or ovarian cancers, most of them involved in HR and Fanconi Anemia pathways [54]. Cancer patients who carry a germline mutation in one of these genes often have other relatives also diagnosed with cancer, triggering genetic screening. However, studies conducted in prostate cancer patients [3,4] have revealed that 30% of patients harboring a germline mutation in a DDR gene and 15% of those who carry a *BRCA2* mutation do not have a relative affected by cancer. Although some reports suggest that intraductal histology may be common in patients with germline *BRCA2* mutations [55,56], no tumor features have been strongly associated with the presence of *BRCA* mutations in prostate cancer beyond a Gleason score >8 and a higher prevalence of node and distant metastasis at diagnosis [8]. Accordingly, updated National Comprehensive Cancer Network clinical practice guidelines [24] now recommend clinicians consider germline screening for mutations in *BRCA2* and other HR genes in all patients with high risk localized prostate cancer and metastatic disease. Identification of a germline mutation in a prostate cancer patient would not only have implications for the patient, but should also be followed by genetic testing of all related family members, providing the opportunity for early cancer-specific screening and risk reduction strategies in those found to be carriers [57].

Considering the high prevalence of prostate cancer in developed countries [58], the increasing need for genetic testing, and the shortage of genetic counsellors, it is evident that the traditional approach consisting of pre-test counselling is no longer feasible and new strategies to enable more widespread access to genetic testing in a timely manner are needed. Some institutions are implementing prostate cancer-focused genetic clinics alongside their pre-existing prostate cancer clinics. Under this approach, patients eligible for testing may undergo counselling by a trained urologist or oncologist managing the patient’s prostate cancer [59]. The ENGAGE study recently reported the results of the oncologist-led *BRCA* testing in women with ovarian cancer and demonstrated efficient turnaround times along with high levels of patient and physician satisfaction [60]. This could also be an adequate and efficient strategy to counsel prostate cancer patients before undertaking a genetic test.

## 6. Conclusions and Future Directions

DDR defects are present in prostate cancer at a higher prevalence than previously recognized. *BRCA2* is the DDR gene most commonly mutated in advanced prostate cancer with up to 5.3% of these patients carrying a germline mutation. Often, carriers lack a personal or family history of cancer to suspect a heritable mutation, therefore germline screening should be considered in all patients with advanced prostate cancer, at least until we are able to discern in which patients the likelihood of a germline mutation is low enough to spare screening. Understanding the real prevalence of germline DDR mutations in prostate cancer across populations would require further studies into screening groups with different genetic backgrounds. Treating physicians are becoming aware of the clinical implications of identifying these alterations and thanks to the more widespread access to genetic testing, we are likely to obtain an accurate estimation of this prevalence in the near feature. Major efforts are needed to guarantee the carriers’ access to a genetic counsellor in a timely manner as well as to establish management protocols that improve these patient outcomes.

The approval of the PARP inhibitors to treat mCRPC patients with DDR defects is likely to occur in the foreseeable future. Pending questions to be answered by currently ongoing trials are the benefit of using PARP inhibitors at earlier disease stages either in monotherapy or in combination. However, stratification of patients for PARP inhibition therapy by DDR defects is still suboptimal and represents the major hurdle for the development of PARP inhibitors in this population. Each of the ongoing phase II/III clinical trials testing the antitumor activity of PARP inhibitors and each laboratory performing genetic diagnostic tests uses a different panel. Even more importantly, the analyses pipelines and the criteria to classify genetic variants may differ significantly [61]. In addition, loss of function may occur without changes in a gene sequence, but may be driven by other mechanisms including epigenetic and transcriptomic changes. Assessing all the heterogeneous and complex mechanisms that could lead to deficient DDR using the methodologies currently available is not cost-effective and we may be under-identifying patients likely to benefit from PARP inhibitors. Further efforts are needed to ascertain DDR deficiency in a comprehensive and efficient approach.

## Figures and Tables

**Table 1:** DDR genes screened for germline mutations in mCRPC studies mentioned in this review.

Pritchard et al. [3] and Mateo et al. [20]				
ATM	ATR	BAP1	BARD1	BRCA1
BRCA2	BRIP1	CHEK2	FAM175A	GEN1
MLH1	MRE11A	MSH2	MSH6	NBN
PALB2	PMS2	RAD51C	RAD51D	XRCC2
Castro et al. [4]				
APEX1	ERCC4	GTF2H5	NTHL1	RBBP8
APEX2	ERCC5	KIAA0415	OGG1	RPA1
APLF	ERCC6	LIG4	PALB2	RPA2
ATM	ERCC8	MBD4	PARP1	RPA3
ATR	FAAP20	MLH1	PARP2	SLX1A
BARD1	FAAP24	MLH3	PARP3	SLX1B
BRCA1	FAM175B	MMS19	PMS1	SLX4
BRCA2	FANCA	MNAT1	PMS2	SMUG1
BRIP1	FANCB	MPG	PNKP	TDG
CDK7	FANCC	MRE11A	PRKDC	UNG
CDK12	FANCD2	MSH2	RAD9A	XAB2
CHEK1	FANCE	MSH3	RAD17	XPA
CHEK2	FANCF	MSH4	RAD23A	XPC
DCLRE1C	FANCG	MSH5	RAD23B	XRCC1
DDB1	FANCI	MSH6	RAD50	XRCC2
DMC1	FANCL	MUS81	RAD51	XRCC3
EME1	FANCM	MUTYH	RAD51B	XRCC4
EME2	GEN1	NBN	RAD51C	XRCC5
EPCAM	GTF2H1	NEIL1	RAD51D	XRCC6
ERCC1	GTF2H2	NEIL2	RAD52	
ERCC2	GTF2H3	NEIL3	RAD54B	
ERCC3	GTF2H4	NHEJ1	RAD54L	
Antonarakis et al. [21]				
ATM	CDK12	FAM175A	GEN1	PIF1
ATR	CENPQ	FAM175B	HDAC2	PMS2
BAP1	CHEK1	FANCA	MLH1	RAD51
BARD1	CHEK2	FANCC	MLH3	RAD51B
BLM	EPCAM1	FANCD2	MRE11A	RAD51C
BRAP	ERCC1	FANCE	MSH2	RAD51D
BRCA1	ERCC2	FANCF	MSH6	RAD54L
BRCA2	ERCC3	FNCG	MUTYH	RDM1
BRIP1	ERCC4	FANCI	NBN	TP53
CDH1	ERCC6	FANCL	PALB2	XRCC2
Annala et al. [22]				
ATM	ERCC1	FANCA	FANCG	RAD51B
ATR	ERCC2	FANCC	MLH1	RAD51C
BRCA1	ERCC3	FANCD2	MSH2	
BRCA2	ERCC4	FANCE	MSH6	
CDK12	ERCC5	FANCF	PALB2	

**Table 2:** Ongoing clinical trials evaluating PARP inhibitors in prostate cancer.

Clinical Trial	Phase	PARP Inhibitor	Study Population	DDR Defects Screening	Strategy	Primary Endpoint
NCT02324998 (CaNCaP03)	I	Olaparib	Intermediate/High Risk PCa	✗	Olaparib +/− Degarelix before radical prostatectomy	Determination of PARP inhibition
NCT02861573 (KEYNOTE-365)	I	Olaparib	mCRPC	✗	Cohort A: Pembrolizumab + Olaparib in post-docetaxel setting	PSA50 response rate
NCT03317392	I/II	Olaparib	mCRPC	✗	Ra223 +/− Olaparib in mCRPC patients with bone metastases	MTD of combination and rPFS
NCT03787680 (TRAP trial)	II	Olaparib	mCRPC	✓	Olaparib + ATR inhibitor (AZD6738) in second-line setting	Response Rate
NCT03432897 (BrUOG 337)	II	Olaparib	Locally advanced Prostate Cancer	✓	Olaparib prior to radical prostatectomy	PSA response rate
NCT03012321	II	Olaparib	mCRPC	✓	Olaparib +/− Abiraterone/Prednisone in first-line setting	PFS
NCT03434158 (IMANOL)	II	Olaparib	mCRPC	✗	Olaparib for patients who are responding after docetaxel-chemotherapy	rPFS
NCT03263650	II	Olaparib	AVPC	✗	Olaparib for patients who are responding after cabazitaxel plus carboplatin	PFS
NCT03570476	II	Olaparib	Localized PCa	✓	Olaparib before radical prostatectomy	pCR rate
NCT03047135	II	Olaparib	Biochemically-recurrent High-Risk PCa	✓	Olaparib in biochemically-recurrent prostate cancer	PSA response rate
NCT03516812	II	Olaparib	mCRPC	✓	Olaparib + Testoterone Enanthate in post-abiraterone/enzalutamide setting	PSA50 response rate
NCT01682772 (TOPARP)	II	Olaparib	mCRPC	✓	Olaparib in post-docetaxel setting	Response Rate
NCT02893917	II	Olaparib	mCRPC	✗	Olaparib +/− Cediranib in second-line setting	rPFS
NCT02987543 (PROfound)	III	Olaparib	mCRPC	✓	Olaparib vs. Abiraterone or Enzalutamide in post-ASI setting	rPFS
NCT03732820	III	Olaparib	mCRPC	✗	Abiraterone/Prednisone +/− Olaparib in first-line setting	rPFS
NCT03076203 (NiraRad)	I	Niraparib	mCRPC	✗	Niraparib + Radium-223	MTD
NCT03431350 (QUEST)	I/II	Niraparib	mCRPC	✓	Niraparib + Abiraterone/Prednisone or JNJ-63723283 in post-ARSI setting	Incidence of toxicities and ORR
NCT02854436 (Galahad)	II	Niraparib	mCRPC	✗	Niraparib in Post-docetaxel and post-ARSI setting	ORR
NCT03748641	III	Niraparib	mCRPC	✓	Abiraterone/Prednisone +/− Niraparib in first-line setting	rPFS
NCT03413995 (TRIUMPH)	II	Rucaparib	mHSPC	✓	Rucaparib without ADT (mHSPC without large lymph nodes and visceral disease)	PSA response rate
NCT02952534 (TRITON2)	II	Rucaparib	mCRPC	✓	Rucaparib in Post-docetaxel and post-ARSI setting	ORR
NCT03533946 (ROAR)	II	Rucaparib	nmHSPC	✓	Rucaparib in nmHSPC with PSADT <10 months	PSA50 response rate
NCT03338790 (CheckMate 9KD)	II	Rucaparib	mCRPC	✗	Nivolumab + Rucaparib or Docetaxel or Enzalutamide	ORR
NCT03442556	II	Rucaparib	mCRPC	✓	Rucaparib for patients who are responding after docetaxel plus carboplatin	rPFS
NCT02975934 (TRITON3)	III	Rucaparib	mCRPC	✓	Rucaparib vs. Abiraterone/Enzalutamide/Docetaxel in second-line setting	rPFS
NCT03330405 (Javelin PARP Medley)	II	Talazoparib	Locally advanced or metastatic tumors	✗	Avelumab plus Talazoparib in advanced solid tumors	DLT
NCT03148795 (TALAPRO-1)	II	Talazoparib	mCRPC	✓	Talazoparib in post-docetaxel and post-abiraterone/enzalutamide setting	ORR
NCT03395197 (TALAPRO-2)	III	Talazoparib	mCRPC	✓	Enzalutamide +/− Talazoparib in first-line setting	rPFS

DDR: DNA Damage Repair; PCa: Prostate Cancer; mCRPC: metastatic Castration Resistant Prostate Cancer; AVPC: Aggressive Variant Prostate Cancer; mHSPC: metastatic Hormone Sensitive Prostate Cancer; nmHSPC: non-metastatic Hormone Sensitive Prostate Cancer; Ra223: Radium-223; ARSI: Androgen receptor signaling inhibitor (abiraterone, enzalutamide); ADT: Androgen Deprivation Therapy; PSADT: PSA Doubling Time; PSA50 response: 50% reduction in PSA levels from baseline; MTD: Maximum Tolerate Dose; rPFS: radiographic Progression-Free survival; PFS: Progression-Free Survival; pCR: pathologic Complete Response; ORR: Overall Response Rate; DLT: Dose Limiting Toxicities.
